# The Effect of Electrical Stimulation on the Cellular Response of Human Mesenchymal Stem Cells Grown on Silicon Carbide-Coated Carbon Nanowall Scaffolds

**DOI:** 10.3390/bioengineering12101073

**Published:** 2025-10-02

**Authors:** Koki Ono, Ayako Tanaka, Kenji Ishikawa, Wakana Takeuchi, Kenichi Uehara, Shigeo Yasuhara, Masaru Hori, Hiromasa Tanaka

**Affiliations:** 1 Graduate School of Engineering, Nagoya University, Furo-cho, Chikusa-ku, Nagoya 464-8601, Japan; ishikawa.kenji.s1@f.mail.nagoya-u.ac.jp; 2 Center for Low-Temperature Plasma Science, Nagoya University, Furo-cho, Chikusa-ku, Nagoya 464-8601, Japan; tanaka.ayako.x7@f.mail.nagoya-u.ac.jp (A.T.); hori.masaru.g1@f.mail.nagoya-u.ac.jp (M.H.); 3 Department of Electrical and Electronics Engineering, Aichi Institute of Technology, 1247 Yachigusa Yakusa-cho, Toyota 470-0397, Japan; wtakeuchi@aitech.ac.jp; 4 Japan Advanced Chemicals Ltd., 3007-4 Kamiechi, Atsugi-shi 243-0801, Japan; kenichi.uehara@japanadvancedchemicals.com (K.U.); shigeo.yasuhara@japanadvancedchemicals.com (S.Y.)

**Keywords:** carbon nanowalls, silicon carbide, human mesenchymal stem cells, neuronal differentiation, osteogenic differentiation, electrical stimulation

## Abstract

Silicon carbide (SiC)-coated carbon nanowalls (CNWs) have been proposed for use as implantable scaffold electrodes. Therefore, we investigated the effects of the SiC coating on CNWs and assessed the effects of the application of electrical stimulation (ES) on human mesenchymal stem cells cultured on SiC-coated CNWs. Measurements were conducted using immunofluorescence staining, proliferation assays, and quantitative reverse transcription polymerase chain reaction. Our results showed that the SiC coating increased the cell adhesion area, and the combination of the SiC coating and ES promoted cell proliferation. Furthermore, ES enhanced osteogenic differentiation on CNWs, both with and without the SiC coating. In SiC-coated samples, the increase in wall thickness of CNWs by the SiC coating promoted neural differentiation. These findings indicate that scaffold electrodes composed of SiC-coated CNWs enhance cell adhesion and proliferation; the application of ES to such electrodes promotes osteogenic differentiation, while the SiC coating itself promotes neural differentiation.

## 1. Introduction

In regenerative medicine, techniques for inducing cell differentiation are crucial. The differentiation behavior of cells is greatly influenced by the surface properties of the scaffold to which the cells adhere, as well as external stimuli such as electric or magnetic fields. Guvendiren et al. [1]. created hydrogel surfaces with lamellar and hexagonal wrinkle patterns to control the differentiation of human mesenchymal stem cells (hMSCs). Those authors reported that the cells differentiated into an osteogenic lineage when cultured on lamellar patterns, while adipogenic differentiation was observed when cultured on hexagonal patterns. Furthermore, Meng et al. reported that applying a 200 mV electrical stimulus to osteoblasts cultured on a scaffold composed of conductive polypyrrole enhanced the expression of markers of osteogenic differentiation [2]. These findings indicate that the nanoscale structure of the scaffold surface and electrical stimulation (ES) may contribute to cell differentiation. Therefore, material properties (such as nanostructure) and conductivity may serve as highly important factors in the development of cell differentiation techniques.

Carbon nanowalls (CNWs) are vertically oriented multilayered graphene sheets that possess high electrical conductivity and a unique interconnected, maze-like morphology, resulting in a large specific surface area. The exposed graphene edges at the wall tips enable chemical functionalization [3,4,5,6,7,8]. These structural and chemical properties make CNWs promising candidates as cell culture scaffolds for regenerative medicine applications [9,10,11,12,13]. We previously have reported that the combination of ES and the nanostructure of CNWs (fabricated using radical-injection plasma-enhanced (RIPE) chemical vapor deposition (CVD)) has the potential to regulate cell differentiation, depending on the presence or absence of ES [14]. Based on these findings, we are investigating the possible application of CNWs as scaffold electrodes for cell culture. In recent years, regenerative medicine has been the subject of active study, including the potential implantation of cell culture scaffolds into the body and the application of ES (via the scaffold) to promote the growth of stem cells for tissue regeneration and healing. The implantation of electrodes into rats and application of ES has been reported to enhance the expression of neurotrophic factors and to improve the sciatic functional index (SFI) in rat [15]. Another study reported that exposing a critical-sized femoral defect in rats to ES using a scaffold electrode leads to enhanced bone formation, increased expression of bone formation-related genes, and elevated vascular density [16]. Due to the excellent properties of CNWs, they hold promise for applications as a scaffold electrode in vivo. However, graphene, the main component of CNWs, has been reported to exhibit toxicity depending on the material’s size and the presence of functional groups [17], an observation that poses a challenge for biological applications. To address this issue, we have proposed coating CNWs with silicon carbide (SiC) to prevent disintegration of the CNWs in vivo and to shield the exposed functional groups of graphene. SiC is highly attractive as a coating material because of its excellent biocompatibility and mechanical properties, and its potential use as a biointerface also is the subject of active study [18,19,20,21,22,23,24]. However, to the best of our knowledge, the effects on cells of SiC-coated CNWs under ES have not been studied. Previously, our research group reported that SiC-coated CNWs do not negatively affect the growth of osteosarcoma-like osteoblast cells (Saos-2) cultured on the fabricated scaffold [25]. However, when this material is used as an electrode, there is a concern that the ES may cause degradation of the coating material, potentially harming the cells. Furthermore, for applications in regenerative medicine, it is essential to investigate how the combination of SiC coating and ES influences stem cell differentiation.

In the present study, we report on the SiC coating of CNWs for their application as in vivo scaffold electrodes and further investigate the behavior of hMSCs cultured on the fabricated SiC-coated CNWs (SiC/CNWs). CNWs were used as templates to prepare samples with varying wall thicknesses, on which hMSCs were cultured to evaluate the cells’ proliferation and differentiation. hMSCs are multipotent cells that have been studied widely for applications in regenerative medicine. In the present study, we focused on the differentiation of hMSCs into bone and neural lineages, which are commonly investigated for use with scaffold electrode materials.

## 2. Materials and Methods

### 2.1. Deposition of SiC on CNWs

**Figure 1:**
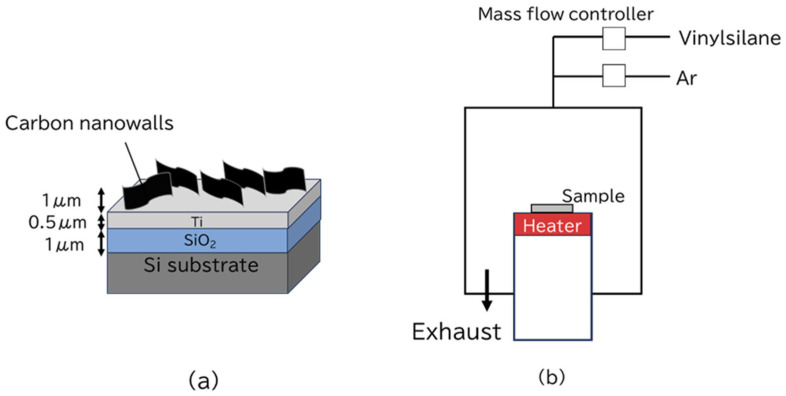
Schematic diagram of (**a**) CNW scaffold and (**b**) thermal chemical vapor deposition (CVD) equipment.

CNWs were deposited on a Ti thin film (500 nm) that was deposited on a SiO_2_ (1 μm)/Si substrate (Figure 1a) by the RIPECVD equipment [26]. SiC was coated onto the CNWs using the cold-wall CVD equipment (Figure 1b). The percentage of CNW edge thickness relative to the total area was calculated by dividing the number of edge pixels by the total number of pixels. The precursor used was vinylsilane (Japan Advanced Chemicals Ltd., Atsugi-shi, Japan). The vinylsilane flow rate was 5 sccm; the diluted Ar gas flow rate was 500 sccm. The surface temperature was 700 °C, as measured by an irradiation thermometer. The SiC deposition time was changed between 5, 15, and 30 min Experimental conditions are shown in Table 1. The samples of SiC-coated CNWs are subsequently referred to as SiC/CNWs. For the purposes of discussion in the present paper, the SiC-coated samples were designated SiC5, SiC15, and SiC30 according to the deposition times.

### 2.2. Evaluation of Surface Morphology of Scaffolds

The morphologies of the CNWs and SiC/CNWs were observed using a scanning electron microscope (Model SU-800, Hitachi-High-Technologies, Tokyo, Japan) equipped with a semi-in-lens detector at an accelerating voltage of 10 kV. The edges and other areas of the CNWs and SiC/CNWs in the scanning electron microscopic (SEM) images were binarized using ImageJ ver 1.54p (National Institutes of Health, Bethesda, MD, USA), and the edge area ratio was calculated at the ratio of number of pixels of the edge to the entire SEM image.

### 2.3. Cell Culture

hMSCs were cultured on the indicated substrate using an acrylic plate, as described previously [22]. Specifically, each CNW or SiC/CNW sample was sandwiched between a bottom and a top acrylic plate. The top plate had an array of through-holes with inner diameter and spacing equivalent to those of a standard 48-well plate. Cells and culture medium were dispensed directly into these holes (hereafter referred to as “wells”), creating isolated culture wells on the exposed scaffold surface. This configuration ensured direct contact between the cells and the substrate. hMSCs were cultured using the medium and supplements supplied in the MSCBM Bullet Kit (Lonza); cultures were propagated at 37 °C in a humidified 5% CO_2_ atmosphere. The control scaffold sample was a 48-well polystyrene cell culture dish. Spent cell culture medium was replaced with fresh medium every 3 days. Where indicated, the cells were subjected to ES one day after seeding. The ES was applied using a function generator with a square wave at an amplitude of 226 mV, a frequency of 10 Hz, and a duty cycle of 50%. These parameters were selected based on our previous studies on osteoblast-like cells cultured on CNWs [14], where a rectangular (square) wave at 226 mV, 10 Hz, and 50% duty cycle effectively promoted cellular activity without cytotoxicity. While previous reports on stem cells commonly describe voltages in the range of 100–500 mV [27,28,29], frequency and duty cycle are not always specified. Therefore, the present choice of 10 Hz and 50% duty cycle represents a moderate and widely used regime in electrical stimulation research.

### 2.4. Immunofluorescence Microscopy

Using a fluorescence microscope (ECLIPSE Ni, Nikon, Tokyo, Japan), a DAPI filter (λex = 358 nm, λem = 468 nm), and a FITC filter (λex = 490 nm, λem = 525 nm), the surface morphology of the cells was observed on the third day, i.e., after the ES had been applied for one day (to cells grown out for one day after seeding). Prior to observation, cells were fixed with 4% paraformaldehyde (062-01661, Fujifilm Wako, Osaka, Japan), permeabilized with Triton X-100 (Sigma, X100, St. Louis, MO, USA), and blocked with normal goat serum (50062Z, Life Technologies, Carlsbad, CA, USA). The cytoskeleton was stained using Actin-Stain 488 phalloidin (PHDG1-A, Cytoskeleton, Inc., Acoma, Denver, CO, USA), and the nuclei then were counterstained using 4,6-diamidino-2-phenylindole (DAPI) (340–07971, DOJINDO, Rockville, MD, USA).

### 2.5. MTS Assay

Cell proliferation was evaluated using the MTS assay, which employs the 3-(4,5-dimethylthiazol-2-yl)-5-(3-carboxymethoxyphenyl)-2-(4-sulfophenyl)-2H-tetrazolium, inner salt (MTS) reagent (Promega Corp., Madison, WI, USA). Briefly, cells were seeded at 1 × 10^4^ cells/well. At 5 days, the culture medium was replaced with culture medium supplemented with MTS reagent at a 1:9 ratio. After 1 h of incubation, the MTS-containing medium was transferred to the individual well of a 96-well plate and light absorbance at a wavelength of 490 nm was measured using a plate reader (Synergy HTX Multi-Mode reader, Bio Tek Instruments, Inc., San Diego, CA, USA). Measurements were performed in four replicates (*n* = 4).

### 2.6. qRT-PCR

The levels of differentiation-related transcripts were determined using the quantitative reverse transcription-polymerase chain reaction (qRT-PCR). The detected mRNAs included those encoding related transcription factor 2 (*RUNX2*), osteocalcin (*OC*), microtubule associated protein 2 (*MAP2*), and glial fibrillary acidic protein (*GFAP*). The transcript encoding the housekeeping protein glyceraldehyde-3-phosphate dehydrogenase (*GAPDH*) was used as a loading control. For this assay, cells were seeded at approximately 7 × 10^4^ cells/well onto a culture surface area equivalent to that of a 48-well plate and cultured for 10 days under the conditions specified in each experiment. Cells then were harvested, and total RNA was extracted using the RNeasy Mini Kit (QiaGen, 74104) and QIA shredder (QiaGen, 79654) according to the manufacturer’s instructions. The total RNA concentration was measured using a NanoDrop One (Thermo Fisher, ND-ONE-W). An aliquot (1 μg) of the total RNA was converted to cDNA using Omniscript Reverse Transcriptase (QiaGen, 205111). The reaction mixture was composed as 3.6 μL RNase free water, 5.0 μL KOD SYBER qPCR Mix (TOYOBO, QKD-201), and 0.2 μL each of 10 μM forward and reverse primers. The sequences of the primer pairs (obtained from Eurofins) were as follows: *RUNX2*, forward 5′ AACCCACGAATGCACTATCCA3′, reverse 5′ CGGACATACCGAGGGACATG 3′; *OC*, forward 5′ TAGTGAAGAGACCCAGGCGC 3′, reverse 5′ CACAGTCCGGATTGAGCTCA 3′; *MAP2*, forward 5′ TGAAGAACATCCGCCACA 3′, reverse 5′ CTTGACATTACCACCTCCAG 3′; *GFAP*, forward 5′ GAGATGCGGGATGGAGAG3′, reverse 5′ TAGGGACAGAGGAGGGAG 3′; *GAPDH*, forward 5′ CGCTCTCTGCTCCTCCTGTTC 3′, reverse 5′ ATCCGTTGACTCCGACCTTCAC 3′. Real-time PCR was performed using a LightCycler (Roche Diagnostics). The 10 μL reaction mixture, distributed to each well of a 96-well plate, consisted of 9 µL of a mixture containing primers and reaction enzyme along with 1 µL of cDNA template. The reaction protocol included an initial denaturation at 98 °C for 2 min, followed by 40 cycles of denaturation at 98 °C for 10 s, annealing at 60 °C for 10 s, and extension at 68 °C for 30 s. All assays were performed in triplicate (*n* = 3).

### 2.7. Data Analysis

All values are presented as mean ± standard error of the mean from three replicates. *p* values were calculated using unpaired two-tailed Student’s *t*-test unless otherwise specified.

## 3. Results and Discussion

### 3.1. Surface Observation of the Scaffold

**Figure 2:**
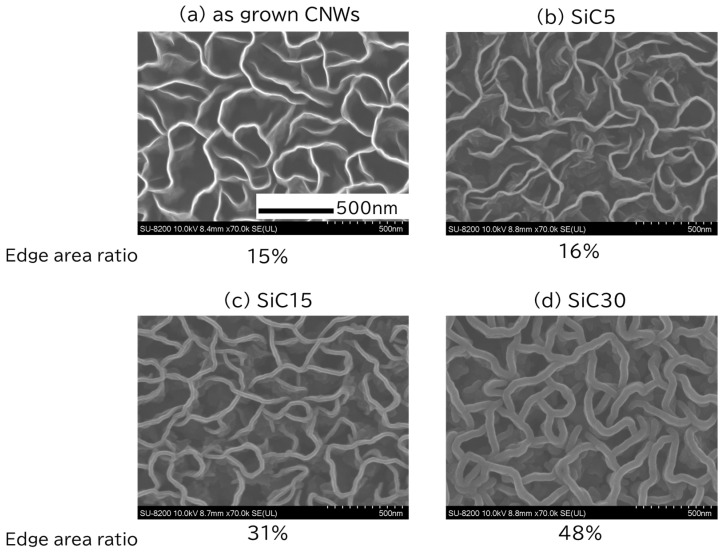
Representative scanning electron microscopy images of Carbon nanowalls (CNWs) and Silicon carbide (SiC)-coated CNWs. (**a**) uncoated CNWs, (**b**–**d**) CNWs coated with SiCs for 5, 15, and 30 min (respectively).

SEM was used to observe the surface morphology of the CNWs and SiC/CNWs. Figure 2 shows representative surface SEM images of CNWs (a) and SiC/CNWs (b–d), with the SiC/CNW images representing CNW coated with SiC for 5, 15, and 30 min, respectively. The edge area ratios for (a–d) were 15%, 16%, 31%, and 48%, respectively. Examination of these images suggested that SiC coating is achieved while maintaining the high-aspect-ratio structure of CNW through thermal CVD using vinylsilane. In addition, based on previous research findings, the coating on the CNW was confirmed as SiC [22].

### 3.2. Immunofluorescence Staining

**Figure 3:**
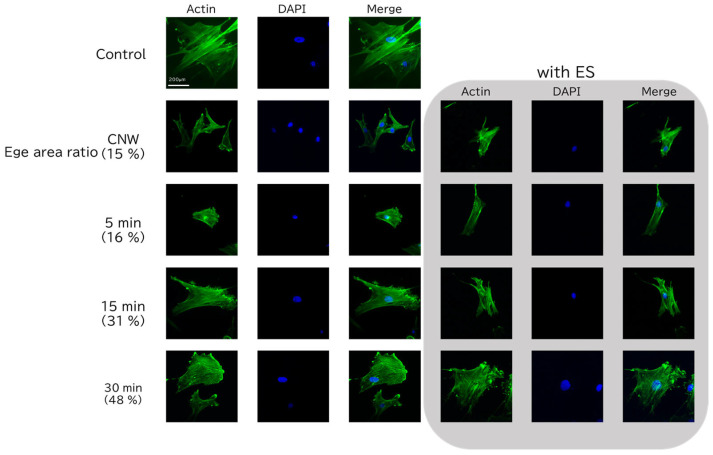
Representative fluorescence microscopy images of cells cultured on carbon nanowalls (CNWs) and silicon carbide (SiC)/CNWs, with or without electrical stimulation (ES). The first column shows actin staining (green) indicating the cytoskeleton; the second column shows DAPI staining (blue) indicating the cell nuclei; and the third column shows merged images of the first and second columns.

Immunofluorescence staining was performed to examine the morphology of cells adhered to CNW and SiC/CNW scaffolds. Figure 3 presents fluorescence microscopy images of cells cultured on culture dishes, CNWs, or SiC/CNW substrates; nuclei and cytoskeletons were stained with DAPI and actin, respectively. Representative images are shown for each substrate, including DAPI staining, actin staining, and merged views. Cells cultured on CNW and SiC5 exhibited limited spreading compared with those on culture dishes, whereas cells cultured on SiC15 and SiC30 displayed cell areas comparable to those on culture dishes. These results indicate that the mean cell area increased with higher edge area ratios, with SiC30 exhibiting a cell area similar to that of the culture dish. No differences in cell area were observed between samples with and without ES. These findings suggest that ES has little influence on cell adhesion, whereas an increased edge area ratio promotes cell attachment and spreading.

### 3.3. Cell Proliferation Assay

**Figure 4:**
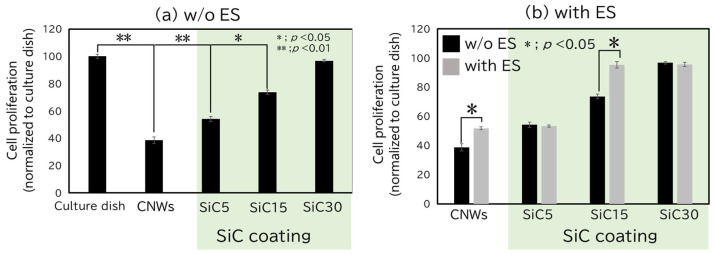
MTS assay results after 5 days of culture. All values are presented as mean ± standard error of the mean (SEM, *n* = 4). *p* values were calculated using Student’s *t*-test. If no asterisk is shown, the result is not significant (NS). (**a**) Comparison of cell proliferation for cultures propagated on culture dishes, CNW, or SiC/CNWs without electrical stimulation (w/o ES). (**b**) Comparison for cultures propagated on CNW and SiC/CNW with and without ES.

To assess cell proliferation, an MTS assay was performed. In Figure 4a, statistical comparisons were performed between the culture dish and all samples under the w/o ES condition. In Figure 4b, CNW w/o ES was used as the reference, and statistical comparisons were conducted with CNW with ES, as well as with SiC5, SiC15, and SiC30 under both with ES and w/o ES conditions. Figure 4a shows the proliferation of hMSCs seeded on substrates consisting of culture dishes, CNWs, and SiC/CNWs after 4 days of culturing without ES. Cells grown on the culture dish exhibited the highest cell viability. However, cells grown on CNWs coated with SiC exhibited enhanced cell proliferation. Notably, in the SiC30 sample, which had the longest coating duration, cell proliferation was comparable to that observed on the culture dish. Additionally, cell proliferation nominally increased with SiC coating duration, corresponding to higher edge area ratios. Figure 4b compares the proliferation of hMSCs on substrates consisting of CNWs and SiC/CNWs, as assessed both with and without ES. For both the CNW and the SiC15 samples, cell proliferation was significantly increased upon ES application. Moreover, under ES conditions, the SiC15 sample showed significantly higher proliferation than the CNW sample. These results suggested that both ES and edge area ratio influence hMSC proliferation, with the effect of ES being more pronounced at lower edge area ratios. ES enhanced cell proliferation for cells cultured on either CNWs and SiC/CNWs, and the combination of ES with increased edge area ratio further potentiated cell proliferation. However, no enhancement of cell proliferation was observed in the SiC5 sample upon ES application. We speculate that this may be due to changes in the capacitance of the SiC coating, caused by variations in film thickness, which could have affected the transmission of electrical stimulation to the cells. However, this remains a hypothesis, and further experiments are required to confirm whether the capacitance change is indeed responsible. A previous study using porous collagen scaffolds investigated the relationship between pore size, which determines the scaffold’s surface area, and cell proliferation, and showed that cell proliferation increased proportionally with the surface area [30]. A separate report on the application of direct current (DC) ES to stem cells demonstrated that, by Day 7, cell proliferation was enhanced by 30% compared to a non-stimulated culture [27]. In the present study, cell proliferation was enhanced on SiC15 compared to that on CNW, under both ES and non-ES conditions. These results suggested that the increased surface area (resulting from SiC coating) and the application of ES independently contribute to the enhancement of cell proliferation. Overall, Figure 3 and Figure 4 demonstrate that SiC-coated CNWs maintain biocompatibility comparable to standard culture dishes, while also offering additional functionalities through electrical stimulation responsiveness and morphological control. These dual characteristics make them highly promising for applications in regenerative medicine.

### 3.4. Cell Differentiation Assay

**Figure 5:**
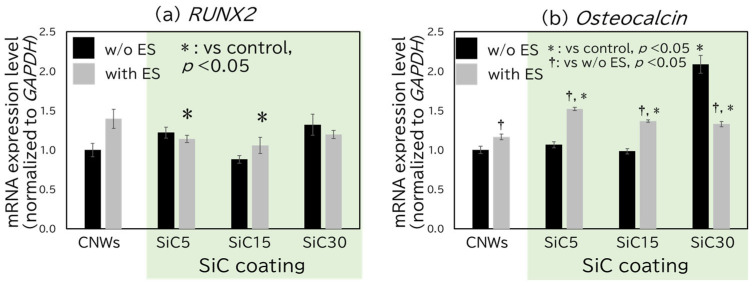
Evaluation of the expression level of osteogenesis marker genes using qRT-PCR, (**a**) *RUNX2*. (**b**) *Osteocalcin*. All values are presented as mean ± standard error of the mean (SEM, *n* = 3). *p* values were calculated using Student’s *t*-test. If no asterisk is shown, the result is not significant (NS).

**Figure 6:**
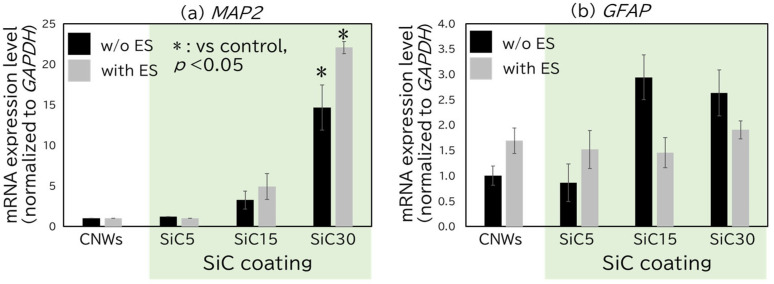
Evaluation of the expression level of neuronal marker genes using qRT-PCR, (**a**) MAP2, (**b**) GFAP. All values are presented as mean ± standard error of the mean (SEM, *n* = 3). *p* values were calculated using Student’s *t*-test. If no asterisk is shown, the result is not significant (NS).

Next, hMSC differentiation was evaluated by determining the levels of transcripts for gene markers associated with development, including *RUNX2* and *OC* for osteogenic differentiation, and *MAP2* and *GFAP* for neurogenic differentiation. *RUNX2* encodes a transcription factor that is essential for osteogenic differentiation; this transcript is considered a key marker of this process. *OC* constitutes a late-stage marker expressed during osteogenic differentiation. *MAP2* is expressed over a broad range of the neuronal differentiation process, from the mid to late stages. *GFAP* is considered a typical differentiation marker for glial cells. In Figure 5 and Figure 6, CNW (w/o ES) was used as the reference, and statistical comparisons were made with all other samples under both w/o ES and with ES conditions. In addition, statistical comparisons were performed between the w/o ES and with ES conditions for each scaffold. Figure 5a shows the expression levels of *RUNX2* after 10 days of culture. Although significant differences were observed between the SiC5 with ES and SiC15 with ES samples and the control, no clear trend was observed across all samples. Figure 5b shows the expression levels of *OC* after 10 days of culture. For cells grown on CNWs, SiC5, and SiC15 substrates, the application of ES significantly potentiated *OC* expression levels. However, for the cells grown on the SiC30 substrate, *OC* expression was significantly attenuated upon the application of ES significantly. The lack of significant changes in *RUNX2* transcript levels in response to ES or changes in edge thickness may reflect the fact that *RUNX2* is an early marker of osteogenic differentiation, the expression of which may have concluded by Day 10 [31,32]. The accumulation of OC mRNA observed after 10 days of ES application is thought to be attributable to the promotion of calcium ion influx. It has been reported that ES induces the influx of calcium into cells [28]. The influxed calcium binds to calmodulin (CaM), thereby facilitating an interaction with Ca/CaM-dependent protein kinase 2 (CaMK2). This interaction enhances the phosphorylation of the osteoblast-specific transcription factor Osterix, which in turn is considered to increase the expression of OC, a differentiation-related gene [33]. Additionally, scaffolds made of cryogels with fine microstructures, *OC* expression has been shown to be increased on substrates with larger specific surface areas [34]. Together, these findings suggest that the accumulation of *OC* transcript observed in cells grown on SiC30 may be attributable to the increased cell adhesion area. The suppression of osteogenic differentiation by ES has been reported in Ref. [12], which demonstrated that ES reduced the expression of RUNX2 and OC in osteoblast-like Saos-2 cells cultured on CNWs. This finding contrasts with those of refs. [30,31], where ES was reported to promote osteogenic differentiation. Notably, the scaffolds used in those studies were flat conductive polymer substrates, suggesting that the effect of ES may vary depending on the physical characteristics of the scaffold. In our study, ES promoted osteogenic differentiation of hMSCs on the sample with the thinnest CNW edge walls, while it suppressed differentiation on the sample with the thickest walls. These results suggest that the effect of ES on osteogenic differentiation of hMSCs may depend on changes in the cell–substrate contact area. In another study where ES was applied to hMSCs, an increase in OC expression and calcium influx was observed after 7 days [29]. ES is thought to enhance osteogenic differentiation by increasing intracellular calcium levels. The elevated calcium activates the CaM signaling pathway, in turn promoting the expression of osteogenic markers.

Figure 6a shows the expression levels of *MAP2* after 10 days of culture. For cells grown on culture dishes, CNWs, and SiC5, the crossing point (Cp) value was 35, indicating that *MAP2* gene expression was nearly undetectable. The Cp value refers to the threshold of the PCR signal. In the SiC15 and SiC30 samples, *MAP2* expression was observed both with and without ES. In the absence of ES, *MAP2* expression levels increased with the edge area ratio. Although no significant differences were observed with and without ES in the SiC15 and SiC30 samples, a notable trend of increased accumulation of *MAP2* mRNA was seen with ES. These results suggested that both ES and increased edge area ratio promote *MAP2* expression; the combination of ES and increased edge area ratio may further enhance *MAP2* expression. Figure 6b shows the expression levels of *GFAP* after 10 days of culture. No significant differences were observed with or without ES or by comparison to cells grown on culture dishes. However, focusing on the mean values, under conditions without ES, *GFAP* expression was increased in SiC15 and SiC30 compared to CNW and SiC5. These results suggest that an increase in edge area may promote *GFAP* expression. Given these results, the results of MAP2 and GFAP expression suggest that an increase in edge area promotes neural and glial differentiation. Yang et al. [35]. reported that the human bone marrow-derived MSCs (hBM-MSCs) cultured on wrinkled structures (with widths ranging from 541 to 3073 nm; as deposited on polydimethylsiloxane scaffolds) exhibited increased *MAP2* expression compared to that seen for cells grown on flat scaffolds, with the accumulation of *MAP2* transcripts increasing with wrinkle width. Those results are consistent with the trend toward increased *MAP2* expression that we observed for cells grown on the SiC15 and SiC30 scaffolds. Just as an increase in wrinkle width enhanced *MAP2* expression in the study by Yang et al. [35]. the increased width of CNW walls (due to increased SiC coating interval) in our experiments appears to have contributed to the accumulation of *MAP2* transcripts.

**Figure 7:**
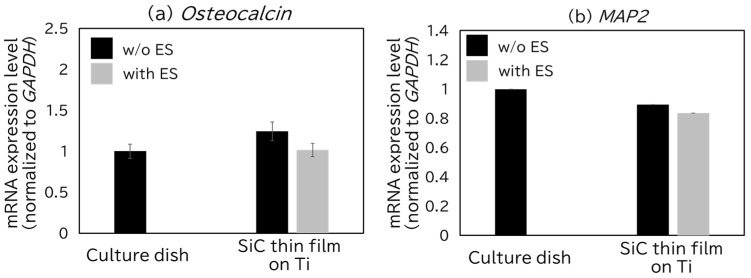
Changes in gene expression levels induced by electrical stimulation (ES) on a flat SiC film using qRT-PCR: (**a**) *Osteocalcin*, (**b**) *MAP2*. All values are presented as mean ± standard error of the mean (SEM, *n* = 3). *p* values were calculated using Student’s *t*-test. If no asterisk is shown, the result is not significant (NS).

To isolate the effect of ES on SiC/CNW substrates, we investigated the influence of ES on cells cultured on a flat SiC thin film. For this purpose, we used a sample prepared by depositing a SiC layer for 15 min using vinylsilane on a 500 nm Ti-coated SiO_2_ (1 μm)/Si substrate. All other deposition and cell culture conditions were identical to those used in previous experiments. The results are presented in Figure 7, the culture dish was used as the control, and comparisons were made with the flat SiC thin film under both conditions. In Figure 7a shows the expression level of *OC*, and Figure 7b shows that of *MAP2*. As shown in Figure 7a, no significant difference in *OC* expression was observed between w/o ES and with ES samples on the SiC surface, and there was also no difference compared to the commercial culture dish. In Figure 7b, the Cp values remained around 35 across all conditions—including the commercial dish and both w/o ES and with ES samples—indicating that MAP2 expression was not detectable. These findings, together with the results shown in Figure 5 and Figure 6, suggest that the effect of ES on cellular differentiation may only become apparent when combined with the physical characteristics of the scaffold.

In summary, while previous studies have reported on osteoblast-like cells cultured on CNWs under electrical stimulation, our study is, to the best of our knowledge, the first to demonstrate the differentiation of hMSCs on SiC-coated CNWs. Furthermore, we show that the combination of SiC coating and electrical stimulation synergistically regulates both osteogenic and neurogenic differentiation, highlighting the unique potential of SiC/CNWs as multifunctional scaffold electrodes for regenerative medicine.

## 4. Conclusions

SiC-coated CNWs were fabricated via RIPECVD and thermal CVD using a vinylsilane precursor, enabling precise increases in the substrate edge area ratio (15% to 48%). hMSC proliferation was significantly potentiated by ES, particularly in samples with a 31% edge area ratio. qRT-PCR analysis revealed that the application of ES resulted in the accumulation of *OC* mRNA without significantly altering *RUNX2* expression, consistent with ES induction of the later stages of osteogenesis. Additionally, *MAP2* transcript levels increased with the edge area ratio, suggesting that the scaffold morphology plays a key role in neuronal differentiation, independent of ES. When ES was applied to hMSCs cultured on a flat SiC thin film, no significant differences were observed in the expression levels of *MAP2* or *OC*, regardless of the presence or absence of ES or the comparison with the control sample. These findings suggest that the effect of ES on gene expression may depend on its combination with the physical properties of the scaffold. These findings highlight the dual influence of ES and CNW architecture on stem cell differentiation, demonstrating the potential of SiC/CNW scaffolds for regenerative medicine applications.

## Figures and Tables

**Table 1:** Condition of SiC coating on CNWs.

Sample Name	SiC5	SiC15	SiC30
Vinylsilane flow rate (sccm)	5		
Ar gas flow rate (sccm)	500		
Surface temp. (°C)	700		
Pressure (Torr)	1		
Growth time (min)	5	15	30

CNWs: Carbon nanowalls, SiC5, 15, 30: Silicon carbide coating, 5 min, 15 min, and 30 min.

## Data Availability

The data that support the findings of this study are available from the corresponding author upon reasonable request.
